# The Production of Vital Force

**Published:** 1849-12

**Authors:** 


### BIBLIOGRAPHICAL NOTICES.

*The Production of Vital Force.*—By Edward Jarvis, M. D. This is the title of an interesting essay read before the Massachusetts Medical Society, at the annual meeting in May, 1849, and published in the transactions of the Society. It embraces much valuable statistical matter relating to the value of life in cities and rural districts compared, its fluctuations at different periods of time, variations in different countries, etc, etc., etc. The object of the author is to invite attention to the laws of health, as involved in the prevention of disease, longevity and offspring. The considerations which he presents, although not original nor novel, are too much neglected, even by medical men, and are practically disregarded, to a greater or less extent, by the larger proportion of every community. To bring the importance of the subject, in its serious aspects and bearings, before the public, is, eminently, the duty of our profession, and it is a duty which has hitherto been inadequately performed. We should be glad to see Dr. Jarvis' essay, in the form of a tract, circulated extensively among persons of all classes, it being adapted to the general, as well as the medical reader.

